# A Rapid Immunization Strategy with a Live-Attenuated Tetravalent Dengue Vaccine Elicits Protective Neutralizing Antibody Responses in Non-Human Primates

**DOI:** 10.3389/fimmu.2014.00263

**Published:** 2014-06-05

**Authors:** Yuping Ambuel, Ginger Young, Joseph N. Brewoo, Joanna Paykel, Kim L. Weisgrau, Eva G. Rakasz, Aurelia A. Haller, Michael Royals, Claire Y.-H. Huang, Saverio Capuano, Dan T. Stinchcomb, Charalambos D. Partidos, Jorge E. Osorio

**Affiliations:** ^1^Takeda Vaccines, Inc., Madison, WI, USA; ^2^Wisconsin National Primate Research Center, University of Wisconsin-Madison, Madison, WI, USA; ^3^Takeda Vaccines, Inc., Ft Collins, CO, USA; ^4^PharmaJet Inc., Golden, CO, USA; ^5^Division of Vector-Borne Diseases, Centers for Disease Control and Prevention, Ft. Collins, CO, USA

**Keywords:** dengue, vaccine, non-human primates, neutralizing antibodies, needle-free delivery, T cell responses

## Abstract

Dengue viruses (DENVs) cause approximately 390 million cases of DENV infections annually and over 3 billion people worldwide are at risk of infection. No dengue vaccine is currently available nor is there an antiviral therapy for DENV infections. We have developed a tetravalent live-attenuated DENV vaccine tetravalent dengue vaccine (TDV) that consists of a molecularly characterized attenuated DENV-2 strain (TDV-2) and three chimeric viruses containing the pre-membrane and envelope genes of DENV-1, -3, and -4 expressed in the context of the TDV-2 genome. To impact dengue vaccine delivery in endemic areas and immunize travelers, a simple and rapid immunization strategy (RIS) is preferred. We investigated RIS consisting of two full vaccine doses being administered subcutaneously or intradermally on the initial vaccination visit (day 0) at two different anatomical locations with a needle-free disposable syringe jet injection delivery devices (PharmaJet) in non-human primates. This vaccination strategy resulted in efficient priming and induction of neutralizing antibody responses to all four DENV serotypes comparable to those elicited by the traditional prime and boost (2 months later) vaccination schedule. In addition, the vaccine induced CD4^+^ and CD8^+^ T cells producing IFN-γ, IL-2, and TNF-α, and targeting the DENV-2 NS1, NS3, and NS5 proteins. Moreover, vaccine-specific T cells were cross-reactive with the non-structural NS3 and NS5 proteins of DENV-4. When animals were challenged with DENV-2 they were protected with no detectable viremia, and exhibited sterilizing immunity (no increase of neutralizing titers post-challenge). RIS could decrease vaccination visits and provide quick immune response to all four DENV serotypes. This strategy could increase vaccination compliance and would be especially advantageous for travelers into endemic areas.

## Introduction

Millions of people living in tropical and subtropical parts of the world are infected with dengue viruses (DENVs) each year (1, 2). The dramatic spread of the disease has been mainly attributed to the geographical expansion of the mosquito vector combined with inadequate measures of vector control, increased human travel, and urbanization (3). DENVs circulate in nature as four distinct serotypes (DENV-1 to DENV-4), each capable of causing a spectrum of disease ranging from subclinical infection to dengue fever (DF), and sometimes to life-threatening dengue hemorrhagic fever (DHF), and dengue shock syndrome (DSS) (1, 4, 5). Normally, infection with one dengue serotype will confer long-term protection against reinfection by the same serotype. However, in case of reinfection by a heterologous dengue serotype, there is the potential risk of antibody-dependent enhancement (ADE) of disease associated with the presence of cross-reactive antibodies (6) and/or cross-reactive T cells (7, 8). Therefore, vaccine development against DENV has focused on tetravalent formulations that can simultaneously provide protection to all four DENV serotypes.

Currently, there are several candidate DENV vaccines at various stages of preclinical and clinical testing (9). This article describes a live-attenuated tetravalent dengue vaccine (TDV) consisting of a molecularly characterized attenuated DENV-2 strain (TDV-2) and three chimeric viruses containing the pre-membrane and envelope genes of DENV-1, -3, and -4 expressed in the context of the TDV-2 genome (TDV-1, TDV-3, and TDV-4, respectively) (10–15). TDV has been extensively tested in preclinical studies (16, 17), two completed Phase 1 clinical trials, and is currently tested in Phase 2 clinical trials in dengue endemic areas. It was shown to be well-tolerated in healthy adults and induced neutralizing antibody responses to all four dengue serotypes (Osorio et al., in preparation; George et al., in preparation).

To improve dengue vaccine delivery globally and in diverse clinical settings an easy delivery method is required combined with a vaccination schedule that will improve compliance. Delivery approaches such as those using jet injectors have been considered as alternatives to the conventional needle and syringe (N–S) injection, with some on the market, and others being tested in clinical trials (18). In this non-human primates (NHP) study, we evaluated the administration of TDV via the subcutaneous (SC) or intradermal (ID) routes using a needle-free delivery device developed by PharmaJet (PhJ). In addition, we investigated rapid immunization strategy (RIS) to administer animals with double doses of vaccine (two separate injection sites, one dose at each site) on day 0 (0, 0). Our results indicated that this RIS strategy induced immune responses comparable to those elicited when two doses are given 53 days apart.

## Materials and Methods

### Viruses and vaccines

DENV-2 New Guinea C (NGC) used as challenge virus in this study was generously provided by Dr. Steven Whitehead (National Institutes of Health, Bethesda, MD, USA). For neutralizing antibody assays, we used virus strains from which the prM and E genes of each live-attenuated dengue vaccine serotype were derived (DENV-1 16007, DENV-2 16681, DENV-3 16562, and DENV-4 1036). DENVs were grown in Vero cells or C6/36 cells in Dulbecco’s modified minimal essential medium (DMEM) containing 5% fetal bovine serum (FBS) and penicillin–streptomycin.

The four vaccine viruses were generated from cDNA clone-derived DENV-2 VV45R virus (based on the genome of DENV-2 PDK-53), and the DENV-2 PDK-53-based chimeras expressing the prM and E genes of DENV-1 16007, DENV-3 16562, or DENV-4 1036. The construction and characterization of these viruses has been previously reported (10, 19).

### Non-human primates

Twelve adult male, DENV seronegative cynomolgus macaques originating from Vietnam were used. The animals were placed in quarantine for 30 days prior to study start. The study was conducted at the Charmany Instructional Facility of the University of Wisconsin, Madison, WI, USA in compliance with the Animal Care Regulations.

### Experimental animal study design

In this study, groups of monkeys (*n* = 3) received the TDV formulated into either 0.5 ml for SC administration or 0.1 ml for ID delivery using the PhJ device. Each full dose of the tetravalent vaccine used in this study contained 2 × 10^4^ PFU of TDV-1, 5 × 10^4^ PFU of TDV-2, 1 × 10^5^ PFU of TDV-3, and 3 × 10^5^ PFU of TDV-4 vaccine viruses. This vaccine constitutes the clinical trial material used for two Phase 1 studies conducted in USA and Colombia, as well as Phase 2 studies currently ongoing in endemic areas.

Each animal in the first two groups received two injections on day 0, one in each arm ID or SC. A third group of animals was injected SC on day 0 and 53 using the PhJ device. Control animals received PBS via the ID route using PhJ. On day 90, all animals were challenged SC with 10^5^ PFU of DENV-2 (NGC strain) using N–S. Serum samples were collected on days 0, 3, 5, 7, 10, 12, 14, 53, 64, 67, and 88 post-primary immunization to analyze vaccine viremia, and days 91, 93, 95, 97, 99, 101, 102, and 104 to analyze DENV-2 NGC viremia after challenge. Serum samples also were collected on days 0, 30, 53, 75, 88, and 104 to determine neutralizing antibody titers to each serotype. PBMCs from group 2 and 4 were collected to measure T cell responses.

### Serum viral RNA

Viral RNA in serum samples was measured using a quantitative reverse transcription-polymerase chain reaction (qRT-PCR) as follows. Viral RNA was extracted from 140 μl of each individual serum sample using a QIAamp viral RNA kit (Qiagen, Valencia, CA, USA) and eluted in 60 μl elution buffer. Viral RNA standards, used to create a standard curve in all qRT-PCR assays, were *in vitro* transcribed from cDNA clones and quantified as previously described (20). E-gene primers, TaqMan probes, and RNA standards were serotype specific (Table 1). Using a different fluorophore for each serotype specific probe (sequences available upon request), qRT-PCRs were performed in duplex: one reaction quantified TDV-1 and TDV-2 vaccine viruses while a separate one quantified TDV-3 and TDV-4 viruses RNA. Following DENV-2 NGC challenge, viral RNA was quantified in a singleplex qRT-PCR. All qRT-PCR reactions were performed in a final volume of 25 μl using the QuantiTect Virus +ROX Vial Kit (Qiagen, Valencia, CA, USA). The reactions contained 5 μl extracted RNA, 0.4 μM of each primer, and 0.2 μM probe. The reaction was conducted in the iQ5 iCycler system (Bio-Rad Laboratories) using the following cycle; 1 cycle of 50°C for 20 min at room temperature (RT), 1 cycle of 95°C for 5 min, and 50 cycles of 95°C for 15 s. Limit of detection for the qRT-PCR was determined for each viral RNA standard by creating a standard curve consisting of nine replicates per dilution. While the sensitivity reached 3.9 copies/reaction (~2.7 log_10_ copies/ml), 3.6 log_10_ copies/ml met the criteria of a 100% detection rate as well as a low ( ≤0.5) cycle threshold standard deviation of the replicates and was used as a cutoff for the assay.

**Table 1:** **E protein primers used in this study**.

	Sequence
**ANTI-SENSE PRIMERS**	
CD1-1593	CAA GGC AGT GGT AAG TCT AGA AAC C
CD2-2116	TCT TAA ACC AGT TGA GCT TCA GTT GT
CD3-2000	CCA CTG GAT TGG CTG TGA TC
CD4-843	GCG CGA ATC CTG GGT TT
**SENSE PRIMERS**	
D1-1459	GACCGACTACGGAACCCTTACAT
D2-1929	TCC ATG CAA GAT CCC TTT TGA
D3-1872	CGC AGC ATG GGA CAA TAC TC
D4-637	GCTGGTGCAATCTCACGTCTA
**PROBES**	
CD1-1519P	CTC GTT AAA ATC TAG CCC TGT CCT AGG TGA ACA AT – FAM
D2-2000P	ACC CAA TTG TGA CAG AAA AAG ATA GCC CAG TC – TET
D3-1914P	AAG ATG CAC CCT GCA AGA TTC CTT TCT C – TET
CD4-699P	TCC GTT CTC CGC TCT GGG TGC AT – FAM

### Microneutralization assay

Serum samples were incubated at 56°C for 30 min to inactivate complement and possible adventitious agents. Heat-inactivated serum samples then were tested for neutralizing activity using a viral immunofocus reduction microneutralization assay and analyzed by an AID ELISpot reader (San Diego, CA, USA). Briefly, 96-well tissue culture plates were seeded with Vero cells at a density of 1.3 × 10^5^ cells/ml in 100 μl/well. Cells were grown at 37°C in a 5% CO_2_ incubator for 48 h. Twofold serial serum dilutions were prepared in a separate 96-well plate and then mixed with virus suspension containing 100 PFU followed by incubation at 4°C for 13–15 h. Culture medium was discarded from the Vero cell plates and then 30 μl of the serum–virus mixture was added to each well in triplicate followed by incubation at 37°C for 2 h. Control positive and negative serum samples were also included. An overlay medium with 1.2% carboxymethyl cellulose was added (100 μl/well) and cells were incubated as above for 2 days for DENV-4, 2.5 days for DENV-1 and -3, and 3 days for DENV-2. After incubation, the overlay was removed and cells were fixed with 85% cold acetone for 10 min at RT. Acetone was then discarded and plates were stored at −20°C until further use. Prior to staining, plates were equilibrated to RT, and washed three times with PBS to rehydrate the cells and to remove any residual overlay. Rabbit anti-DENV polyclonal antibody diluted (1:1000) in PBS-T containing 2.5% (w/v) dry milk powder was added, and plates were incubated at 37°C for 2 h. Plates were washed three times with PBS-T and incubated with anti-rabbit antibody conjugated with horse radish peroxidase (HRP) at 37°C for 2 h. Finally, plates were washed three times with PBS-T and incubated with the substrate (3-amino-9-ethylcarbozole) for 10–30 min or until plaques were visible. The plates were then washed with water and air-dried. The viral immunofoci were quantified on an ELISpot reader. Fifty percent of the average number of foci in the negative control serum defined the cutoff point. The serum dilution closest to the cutoff was recorded as the reciprocal neutralizing titer.

### Intracellular cytokine secretion assay by flow cytometry

To assess the functional capability of TDV-elicited dengue-specific T cells, we performed intracellular cytokine staining (ICS) assays. For positive control, we used Staphylococcus Enterotoxin B (SEB) stimulation, for negative control, we used tissue culture medium devoid of added stimulatory peptides. Peptide arrays used in this study (Table 2) were obtained from the National Institute of Allergy and Infectious Diseases Biodefense and Emerging Infections Research Resources Repository (BEI Resources). Individual peptides were prepared as 10 mM stock solutions for NS1 and NS5 and 15 mM for NS5 peptides. An aliquot of 0.5–1.5 × 10^6^ PBMC in 200 μl total volume was incubated with peptides at 5 μM final concentration in the presence of anti-CD28 (clone L293), anti-CD49d (clone 9F10), and CD107a PE (clone H4A3) antibodies, and 1 μg per test of Brefeldin A (Sigma-Aldrich, St. Louis, MO, USA) and Golgi Stop at 37°C in a 5% CO_2_ incubator overnight. Cells were stained for the surface expression of CD3 (PE-CF594 clone SP34-2), CD4 (PerCP-Cy5.5-conjugated clone L200), CD8 (Pacific Blue-conjugated clone RPA-T8), and live/dead fixable Aqua Dead Cell stain (Invitrogen), washed twice with FACS buffer, and fixed with 2% paraformaldehyde. Cells were then permeabilized with 0.1% saponin buffer, intracellularly stained for IFN-γ (Alexa Fluor 700-conjugated clone 4S.B3), TNF-α (FITC-conjugated clone Mab11), and IL-2 (APC-conjugated clone MQ1-17H12), washed twice with saponin buffer, and fixed with 2% paraformaldehyde. All fluorescent-labeled antibodies and reagents were purchased from BD Biosciences except when mentioned. Sample data were acquired on a SORP BD LSR II equipped with a 50 mW 405 violet, a 100 mW 488 blue, and a 50 mW 640 red laser (BD Biosciences) using FACSDiva version 6.1 acquisition software. We collected approximately 150–300 thousand events in the lymphocyte gate defined by forward and side scatter parameters. Data were analyzed by FlowJo™ 9.4.2 software (Tree Star, Inc., Ashland, OR, USA). Background values from peptide stimulated values were subtracted. The frequency of cytokine-positive T cells was presented as the percentage of gated CD4^+^ or CD8^+^ T cells.

**Table 2:** **Peptide arrays**.

Serotype/peptides	aa. Number	Virus strain	Cat. no. (NBI)
DENV-2 NS1	47	New Guinea C	NR-508
DENV-2 NS3	83	New Guinea C	NR-509
DENV-2 NS5	155	New Guinea C	NR-2746
DENV-4 NS3	106	Singapore/8976/1995	NR-2756
DENV-4 NS5	156	Singapore/8976/1995	NR-4205

## Results

### Vaccine viral RNA following immunization

Following immunization, the presence of vaccine viral RNA in the serum was monitored by qRT-PCR of sequential bleeds collected over a period of 14 days post-primary immunization. TDV induced detectable TDV-2 virus replication from day 5 to 14 for animals injected SC, and day 7–12 for those injected ID (Table 3). No viral RNA from TDV-1, -3, and -4 vaccine viruses was detected in any of the groups on samples collected over a period of 14 days post-primary immunization.

**Table 3:** **TDV-2 virus RNA detected in the serum after primary immunization with TDV**.

Group	Dosing schedule	No. of animals positive for viral RNA				
		Day 5	Day 7	Day 10	Day 12	Day 14
1	TDV PhJ/ID (day 0, 0)	–	1/3	3/3	2/3	–
			(4.8)	(4.5–4.9)	(3.9–4.3)
2	TDV PhJ/SC (day 0, 0)	1/3	3/3	3/3	1/3	–
		(3.8)^a^	(4.0–4.3)	(3.7–4.7)	(4.0)
3	TDV PhJ/SC (day 0, 60)	1/3	3/3	3/3	3/3	2/3
		(3.8)	(4.5–5.4)	(3.8–5.3)	(3.2–4.8)	(3.7–5.0)
4	PBS PhJ/ID (day 0, 60)	–	–	–	–	–

*Results are averages from duplicate or triplicate data*.

*Samples with titers <3.6 log_10_ copies/ml were considered negative*.

*^a^Data in parenthesis represent range of titers in log_10_ copies/ml*.

### Neutralizing antibody responses elicited by vaccination

The individual neutralizing antibody titers and kinetics of antibody responses elicited by TDV are shown in Table 4. Overall, administration of the vaccine by the ID or SC routes using the RIS (0, 0 vaccination schedule) induced comparable neutralizing antibody titers to all four serotypes. In all cases, the dominant neutralizing antibody response was to DEN-2, whereas TDV-4 was the least immunogenic component of the tetravalent vaccine formulation.

**Table 4:** **Kinetics of neutralizing antibody responses in animals vaccinated with TDV SC or ID using the PharmaJet device**.

NHP ID	Vaccine regimen	Day 30				Day 53				Day 88			
		Day 1	Day 2	Day 3	Day 4	Day 1	Day 2	Day 3	Day 4	Day 1	Day 2	Day 3	Day 4
CY0503	0, 0 PhJ/ID	40	1280	160	20	160	1280	40	10	80	5120	20	40
CY0504		80	640	40	10	40	2560	40	5	80	2560	20	40
CY0505		320	1280	2560	320	160	640	640	160	80	640	160	40
	GMT	101	1016	254	40	101	1280	101	20	80	2032	40	40
CY0473	0, 0 PhJ/SC	2560	10,240	160	40	640	5120	80	20	320	1280	40	20
CY0474		1280	320	1280	160	640	640	640	160	320	320	160	80
CY0475		640	320	640	320	640	320	320	80	160	320	160	160
	GMT	1280	1016	508	127	640	1016	254	64	254	508	101	64
CY0493	0, 60 PhJ/SC	160	2560	160	20	80	2560	10	20	320	2560	80	40
CY0494		320	640	640	160	320	640	320	10	80	640	160	80
CY0495		640	2560	320	40	640	2560	40	80	1280	2560	160	40
	GMT	320	1613	320	50	254	1613	50	25	320	1613	127	50

*GMT, geometric mean titer*.

### Characterization of T cell responses elicited by the vaccine

**Figure 1:**
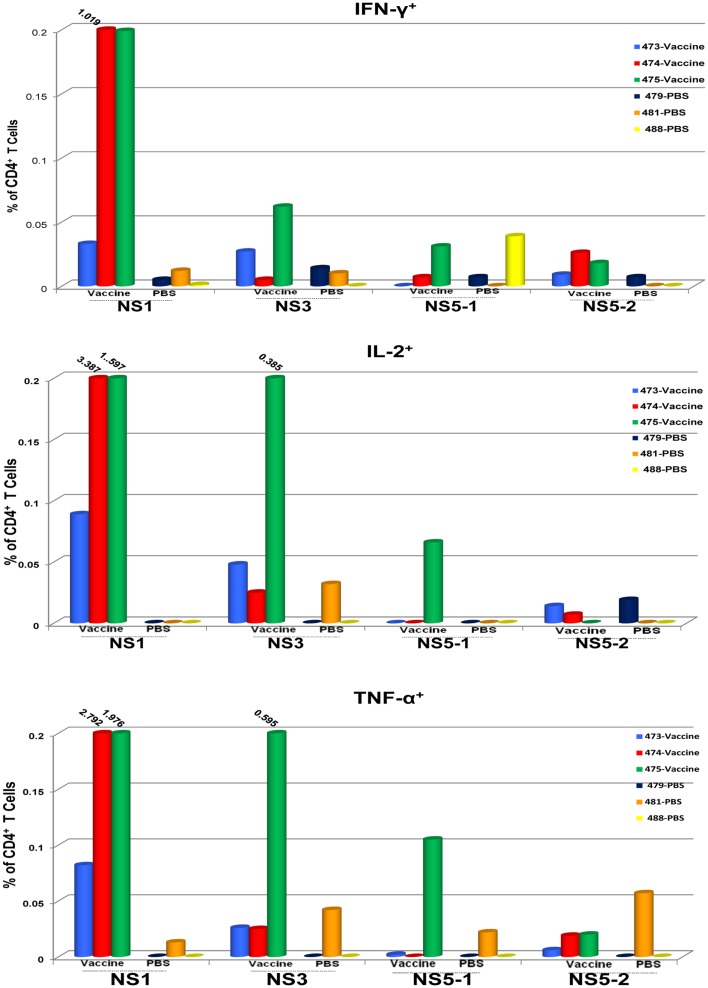
**CD4^+^ T cell responses to TDV target the non-structural proteins of TDV-2**. Responses are shown as percentage of cytokine-positive T cells from DENV-2 peptide arrays stimulated PBMCs with the background percentage of cytokine-positive T cells in medium only treated cells subtracted. Peptide arrays for NS5 were split into two pools; NS5-1 and NS5-2. PBMCs from PBS immunized animals were used as controls.

**Figure 2:**
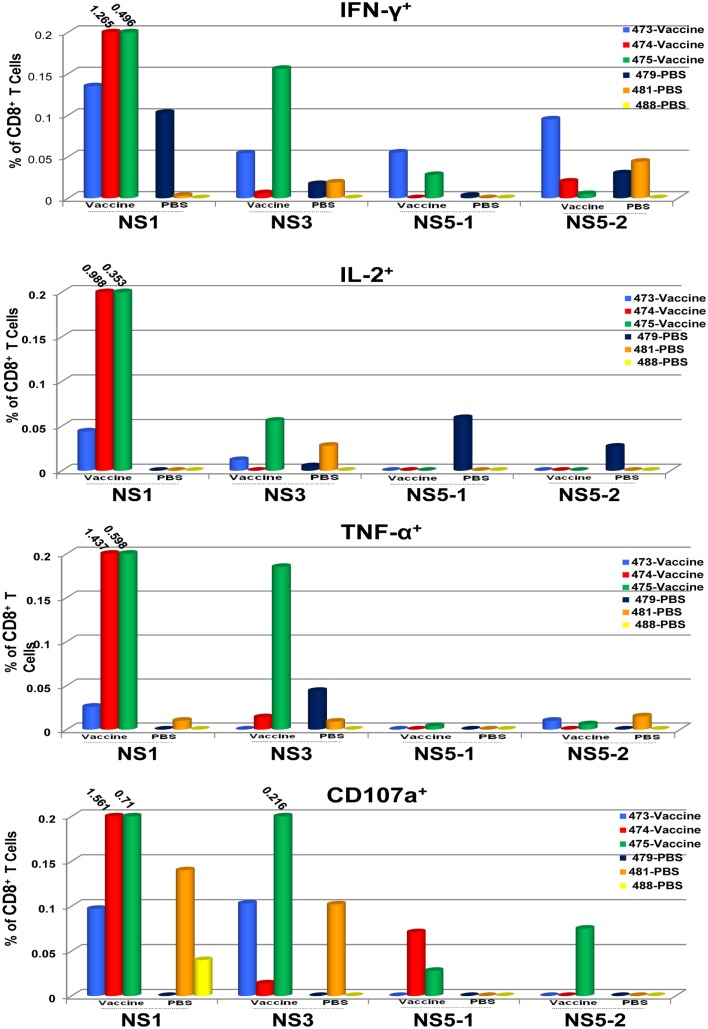
**CD8^+^ T cell responses to TDV target the non-structural proteins of TDV-2**. Responses are shown as percentage of cytokine-positive T cells from DENV-2 peptide arrays stimulated PBMCs with the background percentage of cytokine-positive T cells in medium only treated cells subtracted. Peptide arrays for NS5 were split into two pools; NS5-1 and NS5-2. PBMCs from PBS immunized animals were used as controls.

**Figure 3:**
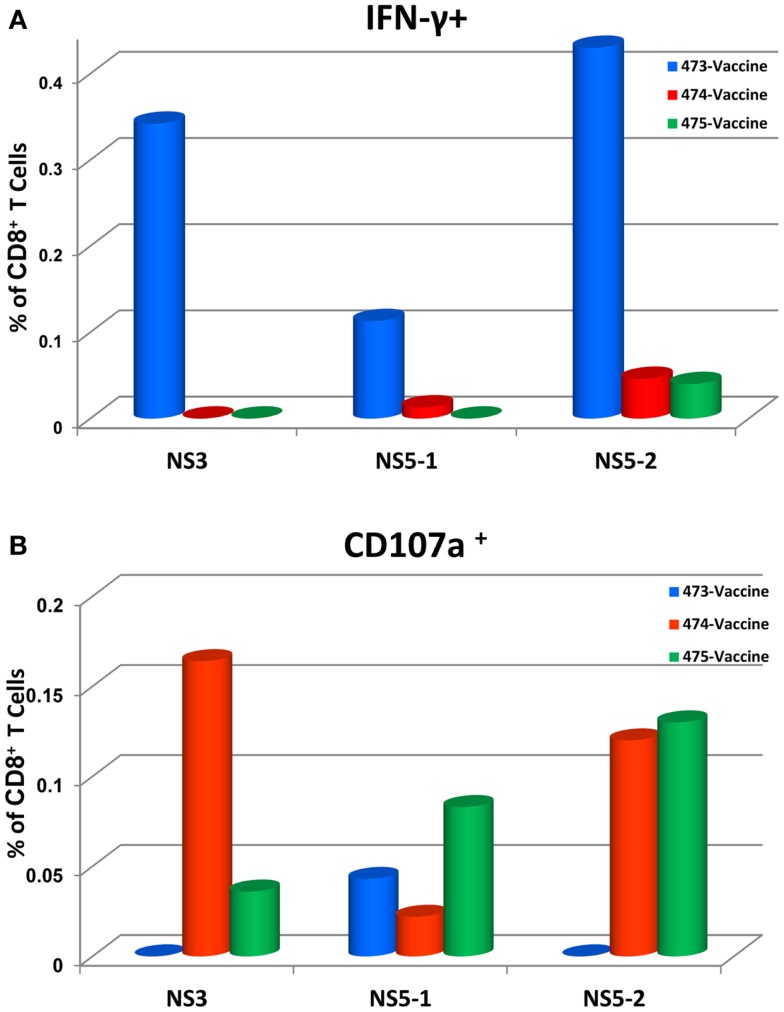
**Tetravalent dengue vaccine elicits CD8^+^ IFN-γ producing T cells that cross-react with NS3 and NS5 proteins of DENV-4 (A) and express the CD107a^+^ marker (B)**. Responses are shown as percentage of T cells from DENV-4 peptide arrays stimulated PBMCs with the background percentage of positive T cells in medium only treated cells subtracted. Peptide arrays for NS5 were split into two pools; NS5-1 and NS5-2. At the time of PBMC testing, peptides arrays for DENV-4 NS1 protein were not available.

To determine the target proteins of the T cell response elicited by TDV, PBMCs from immunized animals (group 2) collected on day 53 post-priming were restimulated *in vitro* with pools of peptides encompassing the entire sequence of DENV-2 NS1, NS3, and NS5 proteins (Table 2). As shown in Figure 1, CD4^+^ T cells predominantly targeted the NS1 protein and to a lesser extent the NS3 and NS5 proteins, producing IFN-γ (a), IL-2 (b), and TNF-α (c). The vaccine also elicited CD8^+^ T cells mainly recognizing epitopes from the NS1 protein and to a lesser degree from NS3 and NS5 proteins (Figure 2). In particular, responses to the NS1 were characterized by the production of IFN-γ (a), IL-2 (b), TNF-α (c), and expression of CD107a^+^ marker (d). In contrast, T cell responses in PBS immunized animals (group 4) were comparatively very low (Figures 1 and 2). In addition, vaccine-specific CD8^+^ IFN-γ producing T cells were cross-reactive with epitopes from the NS3 and NS5 non-structural proteins of DENV-4 (Figure 3A) and were shown to express the CD107a^+^ marker (Figure 3B). A similar pattern of T cell responses recognizing predominantly the NS1 protein with no significant differences in frequencies of CD4^+^ and CD8^+^ T cells were also measured in group 3 (data not shown).

### Protection from DENV-2 NGC challenge

Since TDV-2 constitutes the backbone of TDV in this study, we examined the protective efficacy of this vaccine against challenge with DENV-2 NGC strain. Upon DENV-2 NGC challenge viral RNA was detected in the serum of all mock-immunized animals (Table 4). None of the vaccinated animals displayed DENV-2 NGC RNA. When the neutralizing antibody responses to DENV-2 were compared before and after challenge there was no significant increase in antibody titers in all vaccinated groups suggesting that DENVax elicits sterilizing immunity to DENV-2 (Table 5). In contrast, mock vaccinated animals developed a strong anti-DEN-2 neutralizing antibody response after challenge (Table 5).

**Table 5:** **Protection of TDV vaccinated NHPs from DENV-2 NGC challenge**.

Vaccination regimen	Animal ID	Pre-challenge neutralizing titer (day 88)	Post-challenge neutralizing titer (day 108)	Post-challenge viremia (log_10_ copies/ml)					
			
		Anti-DEN-2 Abs		Day 1	Day 3	Day 5	Day 7	Day 9	Day 11
TDV PhJ/ID (day 0, 0)	CY0503	5120	5120	–	–	–	–	–	–
	CY0504	2560	1280	–	–	–	–	–	–
	CY0505	640	640	–	–	–	–	–	–
TDV PhJ/SC (day 0, 0)	CY0473	1280	2560	–	–	–	–	–	–
	CY0474	320	640	–	–	–	–	–	–
	CY0475	320	320	–	–	–	–	–	–
TDV PhJ/SC (day 0, 60)	CY0493	2560	2560	–	–	–	–	–	–
	CY0494	640	640	–	–	–	–	–	–
	CY0495	2560	2560	–	–	–	–	–	–
PBS PhJ/ID (day 0, 60)	CY0479	5	2560	–	4.8	4.9	4.8	–	–
	CY0481	5	640	–	4.1	4.9	4.9	3.7	4.7
	CY0488	5	1280	–	4.5	5.8	5.2	–	–

## Discussion

To facilitate global dengue prevention and control through effective vaccination, a simple and practical method of administration is highly desirable. This study examined several aspects of vaccine delivery in the NHP model. In particular, we compared immune responses elicited by the SC and ID routes using a needle-free disposable syringe jet injection (DSJI) delivery device and assessed RIS as an alternative to the traditional prime/boost vaccination schedule. Immunization with TDV resulted in the detection of only TDV-2 virus RNA in the serum of vaccinated animals. This is consistent with our previous observations in the NHP model (17). The absence of post-boost viremia in animals that received a prime and booster immunization (0, 60) suggests that priming with the tetravalent vaccine was effective in eliciting immune responses able to reduce and control viral replication upon secondary exposure.

The use of a needle-free DSJI delivery device to administer TDV provided strong evidences suggesting the feasibility of an alternate approach to ID administration. Indeed, measurement of neutralizing antibody responses demonstrated that the vaccine was highly immunogenic. However, responses were unbalanced with anti-DEN-2 neutralizing titers being the highest and those against DEN-4 the lowest. This finding is consistent with previous observations made in preclinical animal models (10, 16, 17) and in Phase 1 clinical trials conducted in the USA and Colombia (Osorio et al., and George et al., in preparation) using N–S delivery with a prime/boost schedule. Since DENV-2 is the most frequent serotype implicated in DHF/DSS in secondary DENV infections (21) it could be argued that immunization with TDV could be advantageous in conferring protection against this serotype. When antibodies induced by the 0, 0 and 0, 53 vaccination schedule by the SC route were compared, the overall titers to all four DENV serotypes were similar. This suggests that the 0, 0 immunization schedule can efficiently prime the immune system for tetravalent responses, which can be sustained at high levels up to 3 months. Therefore, this vaccination schedule could be especially advantageous for travelers in endemic areas.

In the context of vaccination, it is critical to characterize the profile of T cell responses and determine the target proteins of this response. The recent analysis of T cell responses from a large cohort of DENV-infected individuals has highlighted the role of T cells in prevention of development of disease (22). In this study, we demonstrated that SC PhJ delivery of TDV using RIS is effective in inducing CD4^+^ and CD8^+^ T cells. Both T cell subsets produced IFN-γ, TNF-α, and IL-2 highlighting their Th1-type immune profile. In addition, using peptide arrays we demonstrated that they predominantly recognized sequences from the NS1 protein and to a lesser extent from NS3 of DENV-2. Moreover, we observed that the TDV-2 backbone elicited cross-reactive T cell responses to the highly conserved NS proteins of DENV-4. Similarly, we have observed cross-reactivity with the NS proteins of DENV-1 and E proteins of each serotype (data not shown). Overall, these findings highlight the potential of TDV-2 backbone to elicit a broad range of cross-reactive T cell responses to all four DENV serotypes.

The protective efficacy of TDV was assessed against challenge with DENV-2 NGC. All vaccinated animals were protected against DENV-2 NGC as shown by the lack of viral RNA post-challenge, whereas control animals were positive for viral RNA. At the time of challenge, animals from all treatment groups had high levels of anti-DEN-2 neutralizing antibodies (GMT > 300) and their titers were not boosted following challenge. This suggests that the vaccine elicited sterilizing immunity against DENV-2 NGC. Although this study was designed to measure efficacy of TDV using a short-term immunization and challenge protocol, we currently plan to address the longevity of the neutralizing antibody response to vaccination and its impact on protection against challenge with all DENV serotypes. Moreover, the recently published data of the first clinical proof-of-concept efficacy study of a TDV demonstrated safety but only partial efficacy against some but not all DENV viruses, and showed that the standard plaque reduction neutralization test used as the primary immune correlate failed to predict efficacy (23). Therefore, further studies are needed to measure neutralization using different cell substrates (24, 25). Despite the limitations of the NHP model to mimic human disease, efficacy studies based on the presence of viremia as an end point can provide critical information about the protective capacity of candidate DENV vaccines since there are several lines of evidences supporting the view that the severity of disease correlates with increased levels of viremia (26, 27). In conclusion, the delivery of our live-attenuated TDV using the PhJ needle-free DSJI technology has the potential to impact future mass vaccination campaigns.

## Author Contributions

Conceived and designed the experiments: Dan T. Stinchcomb, Jorge E. Osorio, Aurelia A. Haller, Charalambos D. Partidos, and Claire Y.-H. Huang. Performed the experiments: Yuping Ambuel, Ginger Young, Joanna Paykel, Kim L. Weisgrau, Michael Royals, and Joseph N. Brewoo. Managed the NHP facility: Saverio Capuano. Analyzed the data: Yuping Ambuel, Eva G. Rakasz, Kim L. Weisgrau, and Charalambos D. Partidos. Wrote the paper: Charalambos D. Partidos.

## Conflict of Interest Statement

Yuping Ambuel, Ginger Young, Joseph N. Brewoo, Joanna Paykel, Aurelia A. Haller, Dan T. Stinchcomb, Charalambos D. Partidos, and Jorge E. Osorio are affiliated with Takeda Vaccine, Inc., Michael Royals is affiliated with PharmaJet, Inc. The other authors declare no conflicts of interest.
