# Erratum: A Rapid Immunization Strategy with a Live-Attenuated Tetravalent Dengue Vaccine Elicits Protective Neutralizing Antibody Responses in Non-Human Primates

**DOI:** 10.3389/fimmu.2014.00436

**Published:** 2014-09-15

**Authors:** Frontiers Production Office

**Affiliations:** ^1^Frontiers Production Office, Frontiers, Switzerland

**Keywords:** dengue, vaccine, non-human primates, neutralizing antibodies, needle-free delivery, T cell responses

Reason for Erratum:

In Table 4 the text of subheadings was changed due to a typesetting error. This error does not change the scientific conclusions of the article in any way. The publisher apologizes for this error and the correct version of Table 4 appears below.

**Table 4:** **Kinetics of neutralizing antibody responses in animals vaccinated with TDV SC or ID using the PharmaJet device**.

NHP ID	Vaccine regimen	Day 30				Day 53				Day 88			
		D1	D2	D3	D4	D1	D2	D3	D4	D1	D2	D3	D4
CY0503	0,0	40	1280	160	20	160	1280	40	10	80	5120	20	40
CY0504	PhJ/ID	80	640	40	10	40	2560	40	5	80	2560	20	40
CY0505		320	1280	2560	320	160	640	640	160	80	640	160	40
	GMT*	*101*	*1016*	*254*	*40*	*101*	*1280*	*101*	*20*	*80*	*2032*	*40*	*40*
CY0473	0,0	2560	10240	160	40	640	5120	80	20	320	1280	40	20
CY0474	PhJ/SC	1280	320	1280	160	640	640	640	160	320	320	160	80
CY0475		640	320	640	320	640	320	320	80	160	320	160	160
	GMT	*1280*	*1016*	*508*	*127*	*640*	*1016*	*254*	*64*	*254*	*508*	*101*	*64*
CY0493	0,60	160	2560	160	20	80	2560	10	20	320	2560	80	40
CY0494	PhJ/SC	320	640	640	160	320	640	320	10	80	640	160	80
CY0495		640	2560	320	40	640	2560	40	80	1280	2560	160	40
	GMT	*320*	*1613*	*320*	*50*	*254*	*1613*	*50*	*25*	*320*	*1613*	*127*	*50*

GMT* = geometric mean titer.

